# Assessing the impact of Ramadan fasting on COVID-19 mortality in the UK

**DOI:** 10.7189/jogh.11.03060

**Published:** 2021-03-27

**Authors:** Salman Waqar, Miqdad Asaria, Nazim Ghouri, Mehrunisha Suleman, Halima Begum, Michael Marmot

**Affiliations:** 1 Department of Primary Care Health Sciences, University of Oxford, Oxford, UK; 2 Department of Health Policy, LSE, London, UK; 3 Institute of Cardiovascular and Medical Sciences, University of Glasgow, Glasgow, UK; 4 Queen Elizabeth University Hospital, Glasgow, UK; 5 The Health Foundation and Center of Islamic Studies, University of Cambridge, Cambridge, UK; 6 The Runnymede Trust, London, UK; 7 Institute of Health Equity, Department of Epidemiology and Public Health, UCL, London, UK

Many of the 1.8 billion Muslims across the world observe the fast of Ramadan [1], abstaining from all food and drink from dawn to dusk – which can be for up to 19 hours. Whilst people suffering from acute or chronic illness are exempt from fasting, some people with significant comorbidities may still choose to fast, often against medical advice [2].

The peak of the first wave of the COVID-19 pandemic in the UK coincided with the start of Ramadan which took place between April – May 2020 and occurred during a period of lockdown with no communal activities. In the UK where nearly 3 million Muslims fast during Ramadan, the Muslim community has been shown to have disproportionately suffered from the health impacts of COVID-19 over the first wave of the pandemic [3].

Leading up to Ramadan there was concern that observing fasting, with its calorific and water restriction, could either exacerbate or predispose people to COVID-19 and cause harm. A rapid evidence review undertaken prior to Ramadan found no evidence against fasting in healthy individuals [4], which was also supported by interim guidance from the World Health Organization [5]. Clinical guidelines were developed to support patient-centered decisions for those who wished to observe fasting, even for those with chronic disease [6,7].

We explored the impact of Ramadan fasting on COVID-19 mortality using England as a case study. Data were compiled from the Office for National Statistics (ONS) data sets which were publicly available.

We identified 17 local authorities (LAs) in England where the Muslim population as measured at the 2011 census made up at least one-fifth of the population. Muslim populations in these LAs ranged from 20% in Rochdale to 42% in Newham. Apart from 2 LAs (Redbridge and Slough), these LAs were amongst the most deprived quintile of LAs in the country as measured by the Index of Multiple Deprivation 2019.

**Figure:**
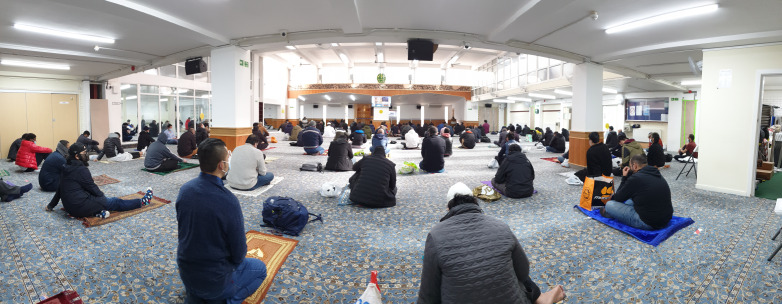
Photo: Socially distanced prayer at Leeds Grand Mosque. Credit: Khalid Abdulla.

We restricted our analysis to these 15 LAs in the most deprived areas in the country to allow us to make a simple comparison against similarly deprived areas with low Muslim populations. The total population across these 15 LAs was approximately 5 million people 1.35 million of whom were Muslim.

A control group of 5 million people was formed by combining the populations of the remaining LAs which were in the most deprived quintile and with the lowest proportions of Muslims in their populations. Muslim populations in this control group of 29 LAs ranged from 0% in Blackpool to 6% in Greenwich.

We aggregated COVID-19 deaths by week and plotted these as rates per 100 000 population for the two groups for weeks 10 through 30 of 2020 both as raw death rates as well as indirectly age-standardised death rates (**Figure 1**). Ramadan began in week 17 and ended in week 21 of 2020.

**Figure 1:**
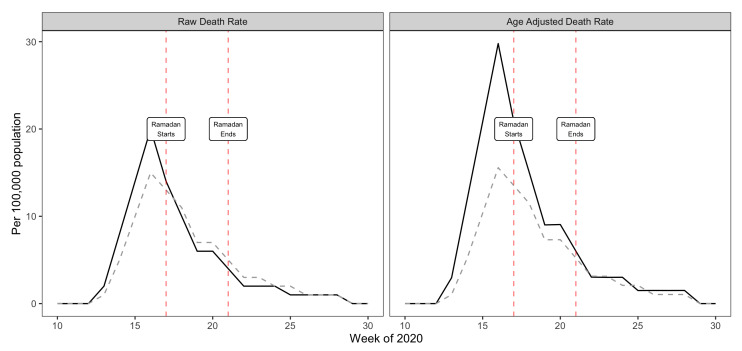
Comparison of COVID-19 deaths in high and low proportion Muslim areas of England during Ramadan 2020. Data for England between week 10 and week 30 of 2020.Data are from ONS (population by religion and COVID-19 deaths). High proportion Muslim areas are deprived Local Authorities with the highest Muslim population (5 million people). Low proportion Muslim areas are deprived local authorities with the lowest Muslim population (5 million people). Full line – high proportion Muslim, dashed line – low proportion Muslim.

The results show that deaths were falling steadily in both Muslim areas and control areas over the Ramadan period. Furthermore, this trend continued in both sets of areas post-Ramadan suggesting that there was no lagged detrimental effect of fasting in the Muslim areas. Age adjusted death rates were higher than raw death rates in the Muslim areas due to their younger than average populations. These age-adjusted death rates suggest that if anything death rates fell faster in the Muslim areas than the control areas over Ramadan albeit from a higher intial peak level (**Figure 1**).

## CONCLUSION

Our findings suggest that the practices associated with Ramadan did not have detrimental effects on COVID-19 deaths. There has been much commentary suggesting that the behaviours and cultural practices of minority communities explain their increased exposure to the pandemic [8]. These claims are not evidence based. Rather, they are unhelpful distractions from inequalities in the social determinants of health, particularly inequalities in living and working conditions, that have been key drivers of health inequalities for all socially disadvantaged groups prior to as well as during the COVID-19 pandemic [9].
